# Examination of the TIGIT-CD226-CD112-CD155 Immune Checkpoint Network during a Healthy Pregnancy

**DOI:** 10.3390/ijms231810776

**Published:** 2022-09-15

**Authors:** Matyas Meggyes, David U. Nagy, Timoteus Feik, Akos Boros, Beata Polgar, Laszlo Szereday

**Affiliations:** 1 Department of Medical Microbiology and Immunology, Medical School, University of Pecs, 12 Szigeti Street, 7624 Pecs, Hungary; 2 Janos Szentagothai Research Centre, 20 Ifjusag Street, 7624 Pecs, Hungary; 3 Institute of Geobotany/Plant Ecology, Martin-Luther-University, Große Steinstraße 79/80, D-06108 Halle, Germany

**Keywords:** TIGIT, CD226, CD112, CD155, pregnancy, immune checkpoint

## Abstract

**Background:** The importance of immune checkpoint molecules is well known in tumor and transplantation immunology; however, much less information is available regarding human pregnancy. Despite the significant amount of information about the TIGIT and CD226 immune checkpoint receptors in immune therapies, very little research has been conducted to study the possible role of these surface molecules and their ligands (CD112 and CD155) during the three trimesters of pregnancy. **Methods**: From peripheral blood, immune cell subpopulations were studied, and the surface expression of immune checkpoint molecules was analyzed by flow cytometry. Soluble immune checkpoint molecule levels were measured by ELISA. **Results**: Notable changes were observed regarding the percentage of monocyte subpopulation and the expression of CD226 receptor by CD4^+^ T and NKT cells. Elevated granzyme B content by the intermediate and non-classical monocytes was assessed as pregnancy proceeded. Furthermore, we revealed an important relationship between the CD226 surface expression by NKT cells and the serum CD226 level in the third trimester of pregnancy. **Conclusions**: Our results confirm the importance of immune checkpoint molecules in immunoregulation during pregnancy. CD226 seems to be a significant regulator, especially in the case of CD4^+^ T and NKT cells, contributing to the maternal immune tolerance in the late phase of pregnancy.

## 1. Introduction

During pregnancy, the maternal immune system undergoes complex and unique developmental events. In the first part of the pregnancy, an inflammatory status is expected to ensure the proper implantation. After that, in the middle of gestation, a symbiotic relationship between the mother and fetus is established, accompanied by a period of rapid fetal growth and development. At the end of the pregnancy, pro-inflammatory immune mechanisms predominate again, playing an important role in the initiation of labor. Proper regulation of the immune homeostasis is needed to sustain these dynamic changes by the immune system in order to promote both pro- and anti-inflammatory biases.

Immune checkpoint receptors and their ligands are crucial for maintaining self-tolerance and regulating immune responses. These receptor–ligand interactions mediate co-stimulatory or co-inhibitory signals in receptor-expressed cells and are essential for proper T cell functioning. After MHC-TCR ligation, stimulatory receptors (CD40, OX-40, CD27, CD137, ICOS) [1,2,3] mediate co-stimulatory signals that provide T cell cytotoxicity and proliferation. T cell inflammatory activity is downregulated through inhibitory immune checkpoint molecules, accompanied by apoptotic mechanisms promoting self-tolerance. Our knowledge about the possible immune checkpoint interactions in reproductive immunology is expanding; however, the data are often controversial [4], and the exact role of these molecules and molecular pathways mediating various biological effects is still unknown.

T-cell Ig and ITIM domain (TIGIT) is a transmembrane glycoprotein that was first published in 2009 [5]. TIGIT is expressed by several immune cell subpopulations, including NK and NKT [6], memory and activated T cells [7], follicular T helper and regulatory T cells [8]. Following the binding with the ligand molecules CD155 (Poliovirus Receptor (PVR or Necl-5)) and CD112 (PVRL2 or Nectin-2) [9], TIGIT mediates inhibitory signaling cascade, which can reduce NK cell cytotoxicity and cytokine production [6] and down-regulate T cell activity [10]. CD226 (DNAM-1) is also a transmembrane glycoprotein on the surface of T cells, NK cells, and monocytes [11]. CD226 is considered a stimulatory checkpoint molecule; it is important in the induction of NK and CD8-mediated immune responses [12]. Since CD226 is a co-stimulatory counterpart of TIGIT, the two surface receptors compete for binding to their ligand. Therefore, the CD155/CD226 interaction transmits an activating signal, whereas TIGIT/CD155 interaction mediates the inhibitory pathway in the cells expressing the receptor [10]. Other interesting data indicate that TIGIT can interfere with the CD226 signaling through the physical prevention of CD226 homodimerization [13].

**Figure 1:**
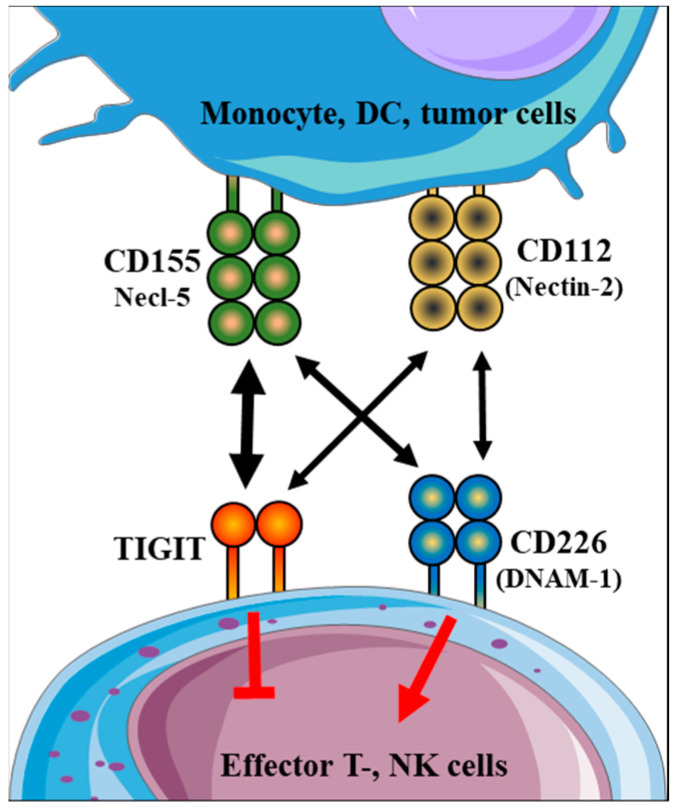
Possible interactions among TIGIT and CD226 surface receptors with CD112 and CD155 nectin and nectin-like molecules. TIGIT and CD226 receptors are primarily expressed on the surface of both T- and NK cells. After interacting with their ligands (CD155, CD112), TIGIT transmits inhibitory signals while CD226 delivers stimulatory signals in the effector cells. The two-sided arrows indicate the possible connections and are proportional to the binding affinities.

As previously mentioned, TIGIT and CD226 can bind CD155 and CD112 ligand molecules (Figure 1). CD155 (Necl-5) and CD112 (Nectin-2) are receptors for poliovirus and members of the nectin and nectin-like family of immunoglobulin superfamily receptors [14]. Publications have shown the surface overexpression of CD155 by various tumor types, including melanoma [15], lung adenocarcinoma [16], pancreatic cancer [17] and soft tissue tumors [18]. Furthermore, our recent study revealed a low level of CD155 expression by various immune cell subsets [19]. While many studies focused on the presence of CD155 on the surface of different tumor types, CD112 expression was detected in diverse cell populations, including endothelial cells, epithelial cells, and fibroblasts [20,21]. It was also observed that the presence of CD112 can correlate with tumor angiogenesis and metastasis [22,23]. Further studies have demonstrated abundant CD112 expression by human DCs derived from monocytes [24], CD14^+^ cells and CD45^−^ cells in various cancers [25,26].

Our scientific knowledge about the possible role of the TIGIT-CD226-CD155-CD112 immune checkpoint network in immunoregulation is emerging. So far, only three publications [19,27,28] have investigated these immune checkpoint molecules in pregnancy. Still, none of them focused on the comparative expression analysis of these molecules in different stages of pregnancy and non-pregnant women.

## 2. Results

### 2.1. Distribution of Peripheral Lymphocyte and Monocyte Subpopulations throughout Healthy Pregnancy and in Non-Pregnant Women

**Figure 2:**
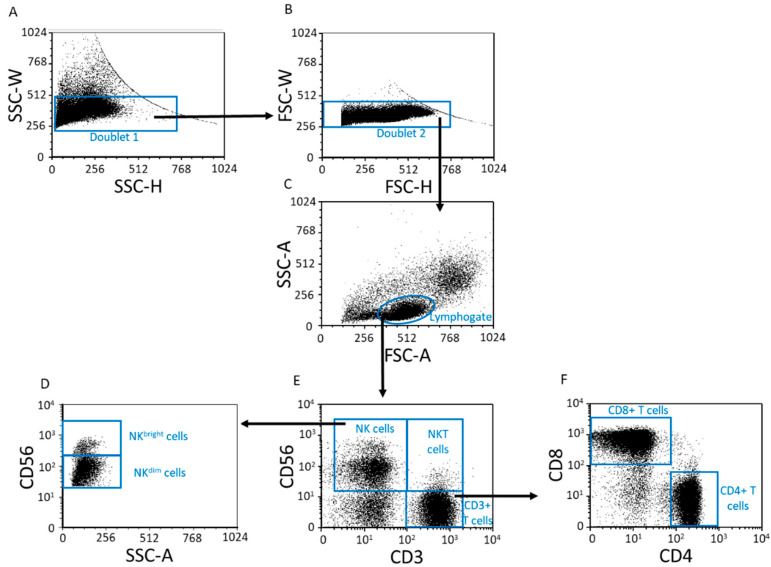
Flow cytometric analyses for determination of lymphocyte subpopulations. Following a two-step doublet exclusion (**A**,**B**), the lymphocyte population was gated using FSC-A/SSC-A parameters (**C**). From the lymphogate CD3^+^ T-, NK-, NKdim, NKbright-, NKT-, CD8^+^ T-, and CD4^+^ T cell subpopulations were detected (**D**–**F**).

Based on the gating strategy, CD3^+^ T, CD4^+^ T, CD8^+^ T, NK, NKdim, NKbright and NKT lymphocyte subpopulations were determined (Figure 2), and their frequencies were compared among the investigated groups (Table 1). Within the lymphocyte population, the only observed significant difference was in the data for CD4^+^ T cells between the third trimester of pregnancy and the non-pregnant state (Table 1).

**Figure 3:**
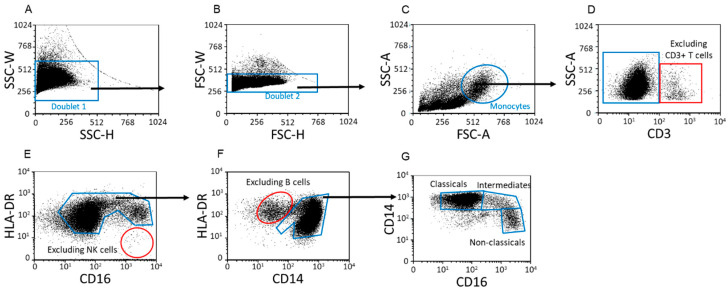
Flow cytometric analyses for determination of monocyte subpopulations. After a two-step doublet exclusion (**A**,**B**), the monocyte population was gated using FSC-A/SSC-A parameters (**C**). From the gated monocytes, the CD3^+^ T cell population was excluded (**D**). CD16 vs. HLA-DR dot plot: CD16^+^/HLA-DR^-^ NK cells could exclude from the monocytes (**E**). CD14 vs. HLA-DR dot plot: HLA-DR high/CD14 low B cells could exclude from the monocytes (**F**). Using CD16 and CD14 markers, monocytes were gated based on their characteristic “┐” shape and subpopulations were differentiated (**G**).

**Figure 4:**
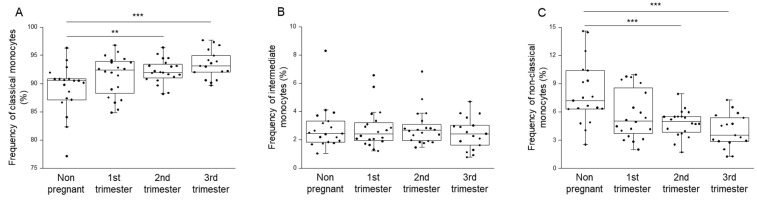
Frequency of different monocyte subpopulations throughout pregnancy and in non-pregnant women. The frequency of classical (**A**), intermediate (**B**), and non-classical monocyte (**C**) cell populations in the peripheral blood during healthy pregnancy and in non-pregnant women. The solid bars represent medians, the boxes indicate the interquartile ranges, and the lines show the most extreme observations. Differences were considered statistically significant for *p*-values <0.01 *** and <0.03 **.

Classical, intermediate and non-classical monocyte subpopulations were determined by flow cytometric analysis (Figure 3), and their distribution was compared among the four cohorts (Table 1). The percentage of classical monocytes—the most abundant subpopulation—was significantly higher in the second and third trimesters of pregnancy than in non-pregnant conditions (Figure 4A). Conversely, the percentage of non-classical monocyte subpopulation was significantly lower in the same pregnant groups than in healthy controls (Figure 4C).

### 2.2. Immune Checkpoint Receptor Expression by Different Immune Cell Subpopulations throughout Healthy Pregnancy and in Non-Pregnant Women

**Figure 5:**
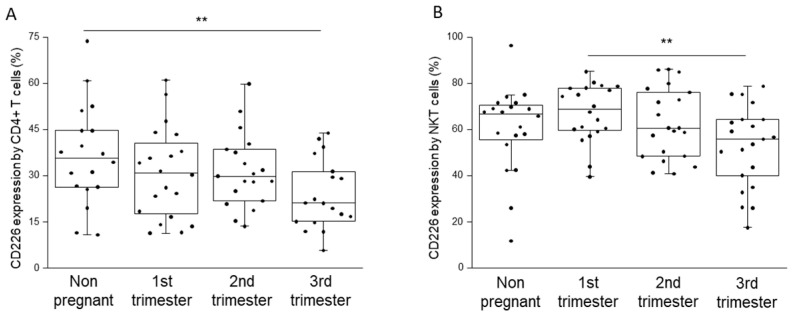
CD226 receptor expression by different T cell subpopulations throughout pregnancy and in non-pregnant women. The expression of CD226 molecule by CD4^+^ T (**A**) and NKT (**B**) cells in the peripheral blood during healthy pregnancy and in non-pregnant women. The solid bars represent medians, the boxes indicate the interquartile ranges, and the lines show the most extreme observations. Differences were considered statistically significant for *p*-values <0.03 **.

There was no significant difference detected, by means of flow cytometric analysis, in the surface expression of the inhibitory TIGIT receptor among all groups involved in our study (Supplementary Material Table S1). The surface expression of their stimulatory counterpart was also measured (Supplementary Material Table S2). The expression of CD226 showed a significant decrease on the surface of CD4^+^ T cells in the third trimester of pregnancy than in non-pregnant state (Figure 5A). Furthermore, a similar decline in the number of NKT cells was measured for the third trimester of pregnancy in comparison to that of the first trimester group (Figure 5B).

### 2.3. Immune Checkpoint Ligand Expression by Different Immune Cell Subpopulations throughout Healthy Pregnancy and in Non-Pregnant Women

**Figure 6:**
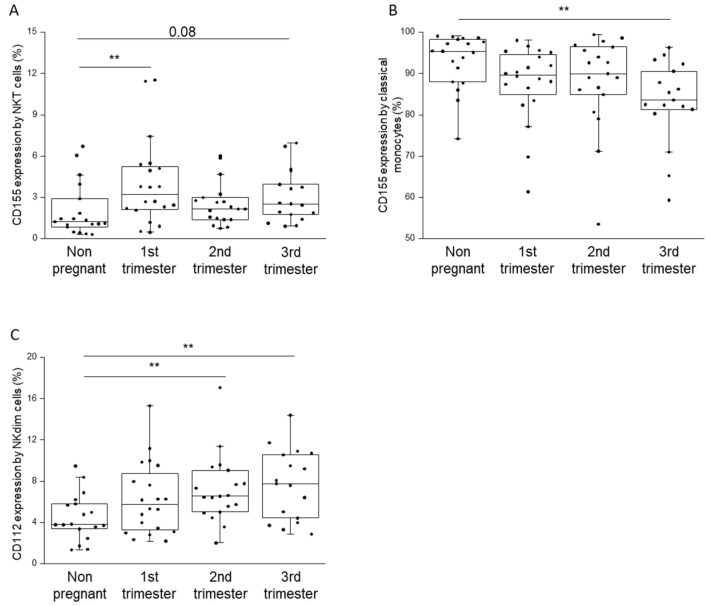
CD155 and CD112 receptor expression by different immune cell subpopulations throughout pregnancy and in non-pregnant women. Significant differences in the expression of CD155 molecule by NKT- (**A**), classical monocyte (**B**) subpopulation and CD112 expression by NKdim (**C**) cell subset in the peripheral blood during healthy pregnancy and in non-pregnant women. The solid bars represent medians, the boxes indicate the interquartile ranges, and the lines show the most extreme observations. Differences were considered statistically significant for *p*-values <0.03 **.

The expression of the immune checkpoint ligands by different immune cell subsets was also examined, and the results were compared among the four cohorts (Supplementary Material Tables S3 and S4). A significantly higher surface expression of the CD155 molecule was detected on NKT cells in the first trimester of pregnancy than on those in the non-pregnant group (Figure 6A). On the contrary, a significantly reduced CD155 presence was detected on the surface of the classical monocyte subpopulation in the third trimester than in the non-pregnant state (Figure 6B). After examining the other ligand molecule, an increasing tendency of the surface expression of CD112 was detected in the NKdim subpopulation during pregnancy, which reached a more significant difference between both the second and third trimesters of pregnancy and the non-pregnant state (Figure 6C).

### 2.4. Comparing the Percentage of TIGIT^−^/CD226^+^ and TIGIT^−^/CD226^−^ Subpopulations throughout Healthy Pregnancy and in Non-Pregnant Women

**Figure 7:**
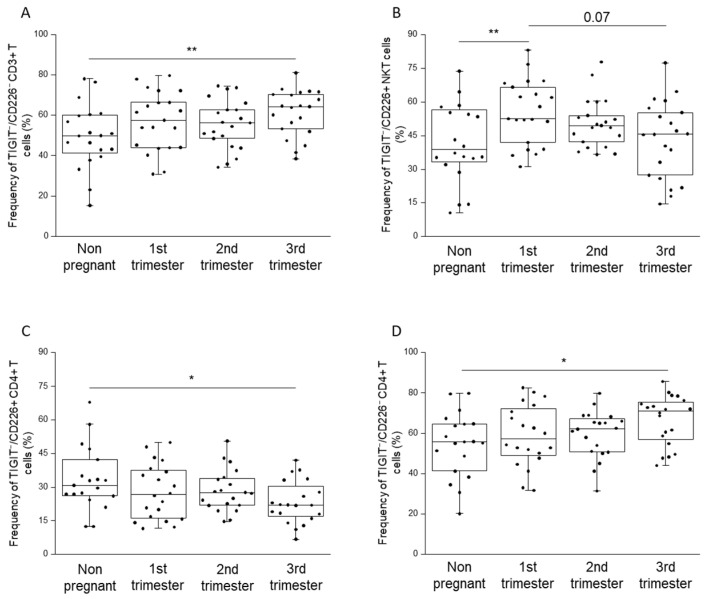
Frequency of TIGIT^−^/CD226^+^ and TIGIT^−^/CD226- cell subpopulations throughout pregnancy and in non-pregnant women. The frequency of TIGIT^−^/CD226^−^ CD3^+^ T cells (**A**), TIGIT^−^/CD226^+^ NKT cells (**B**), TIGIT^−^/CD226^+^ CD4^+^ T (**C**) and TIGIT^−^/CD226^−^ CD4^+^ T cells (**D**) in the peripheral blood during healthy pregnancy and in non-pregnant women. The solid bars represent medians, the boxes indicate the interquartile ranges, and the lines show the most extreme observations. Differences were considered statistically significant for *p*-values <0.03 ** and <0.05 *.

After flow cytometric analyses, we found a significant increase in the percentage of TIGIT^−^/CD226^−^ CD3^+^ T cells in the third trimester of pregnancy compared to those in the non-pregnant group (Figure 7A). A higher percentage of the TIGIT^−^/CD226^+^ NKT cell population in the first trimester of pregnancy than in non-pregnant state was detected. Yet, a higher tendency in the first trimester group compared to the third trimester group was seen (Figure 7B).

Furthermore, a more significant decrease in the ratio of TIGIT^−^/CD226^+^ CD4^+^ T cells of the third trimester of pregnancy than that of in non-pregnant condition was detected alongside a significant elevation of the percentage of TIGIT^−^/CD226^−^ CD4^+^ T cells in the same two groups (Figure 7C,D).

### 2.5. Intracellular Perforin and Granzyme B Content by Immune Cell Subpopulations throughout Healthy Pregnancy and in Non-Pregnant Women

**Figure 8:**
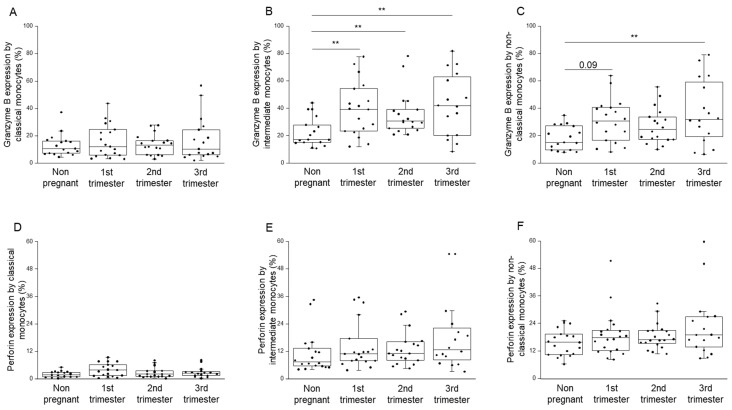
Granzyme B and perforin expression by monocyte subpopulations throughout pregnancy and in non-pregnant women. The intracellular content of the granzyme B (**A**–**C**) and perforin (**D**–**F**) molecule by classical, intermediate and non-classical monocyte subpopulations in the peripheral blood during healthy pregnancy and in non-pregnant women. The solid bars represent medians, the boxes indicate the interquartile ranges, and the lines show the most extreme observations. Differences were considered statistically significant for *p*-values <0.03 **.

Following permeabilization, intracellular perforin and granzyme B content by CD8^+^ T-, NK-, NKdim-, NKbright-, NKT cells, in addition to classical, intermediate, and non-classical monocytes, was measured by flow cytometry. Regarding perforin, we could not detect any significant difference in any monocyte populations among the investigated groups. However, intermediate monocytes showed a significantly increased granzyme B expression in all trimesters compared to that in the non-pregnant state (Figure 8B). Furthermore, the granzyme B content in the non-classical subset in the third trimester of pregnancy was analogously higher than that in the non-pregnant state (Figure 8C). We did not detect any significant difference in the intracellular granzyme B expression by the examined lymphocyte subsets among the investigated groups.

### 2.6. Circulating CD226, CD112 and CD155 Levels throughout Healthy Pregnancy and in Non-Pregnant Women

**Figure 9:**
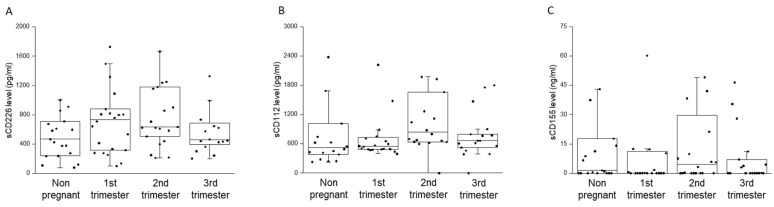
Soluble CD226, CD112 and CD155 molecule levels throughout pregnancy and in non-pregnant women. The serum concentration of sCD226 (**A**), sCD112 (**B**) and sCD155 (**C**) molecules in women during healthy pregnancy and in non-pregnant women. The solid bars represent medians, the boxes indicate the interquartile ranges, and the lines show the most extreme observations.

The concentration of soluble CD226, CD112, and CD155 (sCD226, sCD112, and sCD155) molecules in the sera of pregnant women from all trimesters and of non-pregnant controls was measured by a sandwich ELISA technique (Figure 9). Analyzing the levels of different soluble molecules, we did not find any significant difference among the investigated groups (Figure 9).

### 2.7. Relationship between the Surface CD226 Receptor Expression by CD4^+^T and NKT Cell Subpopulations and the Serum Level of CD226 throughout Pregnancy and in Non-Pregnant Women

**Figure 10:**
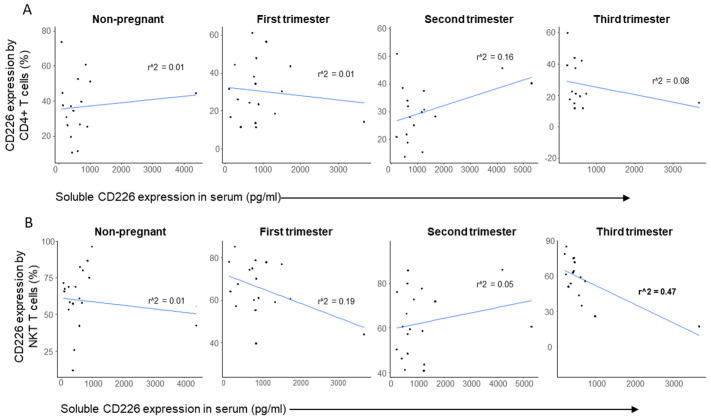
Regression analyses between the surface expression of CD226 and the soluble level of CD226 in CD4^+^ T cells and NKT-like cells throughout pregnancy and in non-pregnant women. Linear regression analyses between the CD226 surface expression and the soluble CD226 level in CD4^+^ T cells (**A**) and NKT-like cells (**B**) in women during healthy pregnancy and in non-pregnant women. *p*-values and coefficients of determination (R2) were calculated in R.

No substantial correlation was found between the surface expression of CD226 by CD4^+^ T cells and the serum sCD226 level (Figure 10A). Nevertheless, we found a significant negative correlation between the expressed and soluble forms of CD226 in the case of NKT cells obtained from women in the third trimester of pregnancy (Figure 10B).

## 3. Discussion

Immune checkpoint molecules and their interaction with the ligands are important in regulating immune responses and ensuring a dynamic balance between tolerance and immunity. In pregnancy, the maternal immune systems must adapt and respond to the presence of the fetus and the presented paternal antigens. Maternal immune tolerance is a complex reaction of the immune system, which has many components in which, based on recent research, immune checkpoint pathways could play a significant role.

Several studies investigated the potential role of TIM-3, CTLA-4, or PD-1 immune checkpoint receptors in healthy and pathological pregnancies [29,30,31,32,33]. Nonetheless, far fewer papers, including ours, examined TIGIT and CD155 and their possible therapeutic value in pre-eclampsia [19,27].

In the case of the phenotypical results, the percentage of the CD4^+^ T cells was more significantly elevated in the third trimester than in the non-pregnant condition, as also observed by Kühnert et al. [34]. However, other examinations, even our previous study [32,33,35], did not find any significant differences in the percentages of the investigated immune cell subpopulations. Contrary to this, Watanabe et al. revealed a notable difference between suppressor T and cytotoxic NK cell numbers between early and late pregnancy [34]. During the phenotypical analyses, a further difference was observed among the monocyte subpopulations; although these data appear controversial, the explanation for the conflicting results lies in the different antibody clones used during flow cytometric investigations [36,37]. According to our data, the increase in the percentage of the classical monocytes during the three trimesters of pregnancy is in line with the decrease in the percentage of the non-classical subset, which emphasizes the role of these subsets in maternal immunoregulation. A high percentage of CD16-expressing monocytes (intermediate and non-classical subset) was detected in several inflammatory conditions and infections [38,39]. Peripheral blood monocytes expressing the low-affinity Fcγ receptor CD16 have been identified previously as a major proinflammatory cell population based on their unique cytokine secretion profile. Therefore, the decreased percentage of the non-classical monocyte subset in the second and third trimester of healthy pregnancy might be a possible factor in the complex maternal immune tolerance toward the fetus. The fact that the granzyme B content is higher in the intermediate and non-classical subsets than in the classical cells supports the pro-inflammatory nature of these cells in middle and late pregnancy. A possible explanation for this might be the inhibited degranulation of these cells, which can protect the placenta or the fetus from the granzyme B mediated proinflammatory effects. At the same time, the elevated granzyme B content by the intermediate and non-classical population, due to the decreased percentage of the non-classical subset in middle and late pregnancy, could be a compensatory mechanism to ensure the immunological defense. Patel et al. published that intermediate and non-classical monocytes arise from the classical monocyte subset [40], which might be restrained in the maternal circulation and could shift the monocytes’ direct pro-inflammatory features to antigen presentation and acquire the CD4^+^ T cell proliferation [41]. Related to this, another paper reported that MHC class I expression was higher by the intermediate and non-classical monocytes, suggesting that these subsets can activate the CD8^+^ T cells [41,42].

Following the phenotypic analysis, notable results were observed regarding the expression levels of the investigated immune checkpoint molecules, especially by the CD4^+^ T and NKT cells. The reduced CD226 expression by the CD4^+^ T cells and the elevated ratio of this subpopulation in the third trimester of pregnancy might be a factor in the maternal immune tolerance. Therefore, CD226 and TIGIT positive and negative subpopulations were further analyzed. The decreased ratio of the CD226^+^ TIGIT^−^ subset, together with the increased percentage of the CD226^−^ TIGIT^−^ subset in the third trimester suggests the importance of the CD226 receptor in late pregnancy. Takahashi et al. published that in patients with cutaneous T-cell lymphoma, the decreased CD226 expression by the CD8^+^ and NK cell populations was accompanied by an increased CD226 serum level compared to healthy controls. Furthermore, they revealed a negative correlation between serum levels of the soluble CD226 molecule and the frequency of CD226^+^CD56^+^ cells among CD56^+^ cells, suggesting that the soluble CD226 molecule originated from the membrane of the NK cells and CD8^+^ cells [43]. According to these findings and our results, we can hypothesize that CD4^+^ T cells potentially lose their CD226 activating receptor by shedding in the third trimester. However, this does not correlate with the serum level of soluble CD226 in this trimester.

The importance of the NKT cells in maternal immune tolerance is well known [44,45,46], even though there is limited data available on the possible role of the TIGIT-CD226-CD112-CD155 immune checkpoint network in reproduction. The high percentage of the TIGIT^−^ CD226^+^ NKT subset in the first trimester compared to non-pregnant status might be part of a Th1 predominance in early pregnancy. As the pregnancy progressed to the third trimester, we detected a decreased ratio and a lower CD226 expression level by these cells, which is inversely correlated with the elevated serum level of soluble CD226. Correspondingly, the CD155 expression by NKT and the CD112 expression by NKdim cells were significantly elevated, indicating a constant TIGIT expression leading to an inhibitory signal during the middle and late pregnancy.

## 4. Materials and Methods

### 4.1. Participants and Sample Collection

Sixty-three healthy pregnant volunteers joined our study in cooperation with the Department of Obstetrics and Gynecology at the University of Pecs. The demographic and obstetrical data of the study participants are presented in Table 2. Twenty-one women from the first, twenty-one from the second and twenty-one from the third trimesters of healthy pregnancies were enrolled. Twenty non-pregnant, healthy, age-matched controls were also recruited by the Regional Blood Transfusion Service of the National Blood Bank. Under the European Union General Data Protection Regulation (GDPR) and Protection of Health and Related Personal Data Act Regulations, all personal and health-related data obtained about the donors during blood donation were processed anonymously, confidentially and securely. They were not provided to the research team for further demographic analysis. None of the recruited women had existing illnesses nor was any of them taking medications. The health status of each of the pregnant women was evaluated, and all women affected by pregnancy-related complications and/or infection, pre-pregnancy disease, in vitro fertilization pregnancies, immune-associated disease, diabetes mellitus or AIDS were excluded. None of the participants were tobacco consumers/smokers.

During sample collection, 20 mL of venous blood was drawn into heparinized tubes, 10 mL of venous blood was taken into plain tubes and immediately transported to the laboratory for further investigation.

### 4.2. Lymphocyte Separation, Cryopreservation, and Thawing

After peripheral blood mononuclear cells (PBMCs) had been separated from heparinized venous blood on density gradient of Ficoll-Paque (GE-Healthcare, Chicago, IL, USA), the PBMC fraction was washed in complete Roswell Park Memorial Institute (RPMI) 1640 medium (Lonza, Switzerland) supplemented with 10% fetal calf serum (FCS, Lonza, Switzerland), counted and centrifuged. For cryoprotection, the cells were resuspended in inactivated human serum containing 10% dimethyl sulfoxide (DMSO) (Sigma-Aldrich, St. Louis, MO, USA), aliquoted in cryovials and stored in a −80 °C mechanical freezer for further investigation. On the day of examinations, the samples were thawed in a 37 °C water bath, resuspended in RPMI-1640 medium, and washed twice to remove the remaining DMSO content.

### 4.3. Flow Cytometric Staining and Analysis

Before labeling, thawed PBMCs were blocked by human Fc receptors using Human TruStain FcX Blocking Solution (Biolegend, San Diego, CA, USA) for 10 min. For flow cytometric labeling, fluorochrome-conjugated monoclonal antibodies (Table 3) were added in various combinations to 10^6^ PBMCs for 30 min at room temperature (RT) in complete darkness. Next, the cells were resuspended in 300 µL of phosphate-buffered saline (PBS) (BioSera, France) containing 1% paraformaldehyde (PFA) and stored at 4 °C in complete darkness until fluorescent activated cell sorting (FACS) analysis. Various flow cytometric analyses were performed with a BD FACS Canto II flow cytometer (BD Immunocytometry Systems, Belgium) with the BD FACS Diva V6. software (BD Biosciences, San Jose, CA, USA) for data acquisition. Flow cytometric data analysis was performed by FCS Express V4 (De Novo Software, Pasadena, CA, USA).

### 4.4. Intracellular Staining

Following surface labeling, cells were washed with PBS and fixed in 4% PFA in PBS for 10 min at RT in complete darkness. After that, the cells were washed with 2 mL of PBS and incubated with a 1:10 diluted FACS Permeabilizing Solution 2 (BD Biosciences) for 10 min at RT in complete darkness. Next, the samples were washed and incubated with FITC-conjugated anti-human granzyme B and PE-Cy7-conjugated anti-human perforin for 30 min at RT in total darkness. Finally, the samples were washed again with 2 mL of PBS, resuspended in 1% PFA, and stored at 4 °C in complete darkness until FACS analysis was performed.

### 4.5. Enzyme-Linked Immunosorbent Assay (ELISA)

To determine the soluble Nectin-2/CD112, DNAM1/CD226 and Necl5/CD155 levels in the serum samples of the participants, sandwich-type ELISA was used according to the protocols provided by the manufacturers.

Nectin-2/CD112 ELISA (Invitrogen^TM^, Thermo Fisher Scientific, EH331RB): Before the assay, frozen serum samples and test reagents were thawed and allowed to reach RT. Then, 100 μL of standards and 2-fold diluted sera were added to the appropriate wells of the test plate precoated with anti-human Nectin-2 antibody. The plate was incubated for 2.5 h at RT with gentle shaking. After washing 4 times with 300 μL/well of wash buffer, 100 μL of biotin-conjugate was added to each well and incubated for 1 h at RT with continuous shaking. Following washing, 100 μL/well of diluted Streptavidin-HRP solution was pipetted to the plate and incubated for 45 min at RT with continuous shaking. Next, the washing step was repeated, and 100 μL of TMB substrate was added to each well. After 30 min of incubation at RT in complete darkness, the color reaction was terminated.

DNAM-1/CD226 ELISA (ELISA Kits: R&D Systems, Bio-Techne, DY666-05, Ancillary Reagent kit 2: R&D Systems, Bio-Techne, DY008): 96-well flat-bottom plates were coated overnight at RT with 100 μL of anti-human CD226-specific capture antibody in PBS. The next day the plate was washed with 3 × 400 μL/well of wash buffer and blocked with 400 μL/well of reagent diluent for 1 h at RT. After washing 3 times, 100 μL of serial-diluted standards and thawed serum samples were added to the plate and incubated for 2 h at RT. Following washing, 100 μL of detection-antibody was pipetted to the wells at the recommended concentration and incubated for a further 2 h at RT. Then, washing was performed 3 times as above and 100 μL/well of streptavidin HRP was added for 20 min in complete darkness. Finally, after 3 washes, the plate was developed for 20 min with a 1:1 mixture of Substrate Reagents A + B in complete darkness, and the reaction was stopped with a Stop Solution.

PVR/Necl5/CD155 ELISA (RayBiotech, Norcross, GA, USA, Cat. No.: EHL-CD155): For quantitative measurement 2.5× diluted standards were prepared freshly. Then, 100 μL of the total of 8-point standards (from 200 ng/mL to 0 ng/mL) and 2-fold diluted sera were added to the appropriate wells of the 96-well plate coated with anti-human CD155 antibodies. The plate was incubated for 2.5 h at RT with gentle shaking. After manual washing with 300 µL of freshly prepared wash buffer 4 times, 100 μL of 1× diluted biotinylated anti-human CD155 antibody solution was added to each well and incubated for 1 h at RT with gentle shaking. After washing 4 times, 100 μL of prepared Streptavidin solution was pipetted into all wells of the plate and incubated for 45 min at RT with shaking. Followed by the 4× final wash, 100 μL of TMB One-Step Substrate Reagent was added to each well and incubated for 30 min at RT in complete darkness with gentle shaking. The color reaction was terminated by the addition of 50 µL of Stop Solution to each well.

The absorbances of the plate wells were read immediately with a SPECTOstar Nano spectrophotometer (BMG Labtech, Ortenberg, Germany) at 450 nm (CD112 ELISA) or 450 nm with a reference filter of 540 nm. After background absorbance subtraction, standard curves were generated by a 4-parametric logistic analysis. The concentrations of soluble Nectin-2 and DNAM-1 in the serum samples were calculated from the standard curve using MARS Data Analysis Software version 3.32 (BMG Labtech, Ortenberg, Germany) and in Necl5 using Microsoft Excel. In the case of CD112 and CD155 ELISA, the obtained concentrations were corrected by the dilution factor 2.

### 4.6. Data Analysis

Our statistical analyses were conducted with linear models using the R environment, testing the effect of trimester on the measured response variables. Decisions on the transformation of variables depended on visual inspection of “model-checking plots” in R for the models with transformed versus untransformed variables. These plots allow checking assumptions about the normality of residuals and variance homogeneity. Response variables related to the immune receptor–ligand relationship were log_e_-transformed with ANOVA used as hypothesis testing, while response variables related to the donors (age, gestational age, gravidity and parity) were analyzed at Poisson distribution, with Chi-square tests as hypothesis testing. Pairwise comparisons were made with Tukey post hoc test.

To test the relationship between the surface expression of CD226 by CD4^+^ T and NKT cells and the soluble level of CD226 in the serum from non-pregnant and pregnant women in all trimesters, linear regression analyses were performed. *p*-values and coefficients of determination (r2) were calculated in R.

## Figures and Tables

**Table 1:** Phenotype characteristics of peripheral blood mononuclear cells throughout pregnancy and in non-pregnant women.

	Non-Pregnant	1st Trimester	2nd Trimester	3rd Trimester	Significant *p*-Values
**CD3^+^ T cells**	61.27 ± 12.54	67.32 ± 9.79	68.44 ± 8.08	69.24 ± 7.62	
**CD4^+^ T cells**	31.11 ± 9.01	36.98 ± 9.80	35.98 ± 7.81	40.45 ± 7.54	<0.01 NP vs. 3rd
**CD4^+^ T cells in CD3^+^ T cells**	50.53 ± 12.14	54.22 ± 9.44	52.71 ± 7.88	58.19 ± 8.45	
**CD8^+^ T cells**	25.30 ± 9.01	24.32 ± 5.65	26.91 ± 4.91	24.27 ± 5.50	
**CD8^+^ T cells in CD3^+^ T cells**	41.18 ± 10.77	36.70 ± 8.95	39.02 ± 7.06	34.97 ± 7.82	
**NK cells**	19.03 ± 9.77	15.36 ± 7.75	12.94 ± 5.18	12.93 ± 5.08	
**NK^dim^ cells**	17.74 ± 9.12	14.00 ± 7.51	11.61 ± 5.16	11.97 ± 4.94	
**NK^bright^ cells**	1.53 ± 2.24	1.36 ± 0.74	1.34 ± 0.58	1.02 ± 0.45	
**NKT cells**	6.84 ± 5.84	5.51 ± 4.17	5.25 ± 3.38	4.83 ± 3.63	
**Classical monocytes**	89.10 ± 4.37	91.35 ± 3.35	92.19 ± 2.13	93.42 ± 2.52	<0.03 NP vs. 2nd <0.01 NP vs. 3rd
**Intermediate monocytes**	2.84 ± 1.56	2.80 ± 1.38	2.87 ± 1.25	2.47 ± 1.13	
**Non-classical monocytes**	7.99 ± 3.32	5.76 ± 2.64	4.82 ± 1.41	3.99 ± 1.77	<0.01 NP vs. 2nd <0.01 NP vs. 3rd

The results were presented as the mean value ± SD. Statistical comparisons were made in R using one-way ANOVA tests. Differences were considered statistically significant for *p*-values ≤ 0.05. NP: Non-pregnant.

**Table 2:** Demographic and obstetrical data of the participants.

	Non-Pregnant	1st Trimester	2nd Trimester	3rd Trimester
**No. of women**	20	26	28	29
**Age (years)**	29.85 (22–43)	32.77 (18–41)	32.54 (18–43)	32.10 (23–44)
**Gestation age at sampling (weeks)**	-	13.96 ± 2.44	24.96 ± 1.82	30.86 ± 3.77
**Gestation age at birth (weeks)**	-	39.05 ± 1.39	38.65 ± 1.87	39.34 ± 0.81
**Mean of gravidity**	-	0.76	0.77	0.89
**Mean of parity**	-	1.15	1.08	1.41

**Table 3:** Fluorochrome conjugated monoclonal antibodies used in the study.

Antigen	Fluorochrome	Clone	Isotype	Company	Cat. No.
**CD112**	PE	R2.525	Mouse IgG1, κ	BD Biosciences	551057
**CD14**	FITC	M5E2	Mouse IgG2a, κ	BD Biosciences	555397
**CD155**	APC	SKII.4	Mouse IgG1, κ	Biolegend	337618
**CD16**	PerCp-Cy5.5	3G8	Mouse BALB/c x DBA/2,	BD Biosciences	560717
**CD3**	BV510	UCHT1	Mouse BALB/c IgG1, κ	BD Biosciences	563109
**CD4**	FITC	RPA-T4	Mouse IgG1, κ	BD Biosciences	555346
**CD8**	APC-H7	SK1	Mouse BALB/c IgG1, κ	BD Biosciences	560179
**CD56**	PerCp Cy5.5	B159	Mouse IgG1, κ	BD Biosciences	560842
**CD56**	APC	B159	Mouse IgG1, κ	BD Biosciences	555518
**CD226**	BV421	DX11	Mouse BALB/c IgG1, κ	BD Biosciences	742493
**Granzyme B**	FITC	GB11	Mouse BALB/c IgG1, κ	BD Biosciences	560211
**HLA-DR**	APC-H7	G46-6	Mouse IgG2a, κ	BD Biosciences	561358
**NKG2D**	PE-Cy7	1D11	Mouse RBF/ DnJ IgG1, κ	BD Biosciences	562365
**Perforin**	PE-Cy7	dG9	Mouse IgG2b, κ	Biolegend	308126
**TIGIT**	PE	A1553G	Mouse IgG2a, κ	Biolegend	372704

## Data Availability

The data presented in this study are available on request from the corresponding author.

## Supplementary Materials

The following supporting information can be downloaded at: https://www.mdpi.com/article/10.3390/ijms231810776/s1.
