# Supplementary material for: Examination of the TIGIT-CD226-CD112-CD155 Immune Checkpoint Network during a Healthy Pregnancy
Source: Int J Mol Sci. 2022 Sep 15;23(18):10776. doi: 10.3390/ijms231810776 (PMC9502426; doi:10.3390/ijms231810776)
Supplement: Supplementary file 1 [file ijms-23-10776-s001.zip › ijms-1872941-supplementary.pdf]

Supplementary Tables

Supplementary Table S1. Surface expression of TIGIT receptor (%) in peripheral blood mononuclear cell populations throughout pregnancy and in non-pregnant women.

	Non-pregnant	1st trimester	2nd trimester	3rd trimester	p-value
CD3+ T cells	22.88±11.03	17.76±6.45	18.86±5.87	17.39±7.55	
CD4+ T cells	11.81±4.91	12.99±4.29	12.02±3.19	10.46±3.92	
CD8+ T cells	33.16±15.23	25.75±9.86	28.80±9.87	27.75±12.33	
NK cells	44.97±18.60	44.53±17.02	49.51±15.55	40.10±15.81	
NK^{dim} cells	46.59±19.07	47.75±17.97	53.21±15.82	42.57±16.52	
NK^{bright} cells	22.01±14.87	16.68±8.33	21.70±12.51	14.83±11.49	
NKT-like cells	36.52±24.75	23.87±16.17	26.80±17.11	29.47±21.59	

Statistical comparisons were made in R using one-way ANOVA tests. The results were presented as the mean value ± SD. No significant differences were observed among the investigated groups.

Supplementary Table S2. Surface expression of CD226 receptor (%) in peripheral blood mononuclear cell populations throughout pregnancy and in non-pregnant women.

	Non-pregnant	1st trimester	2nd trimester	3rd trimester	p-value
CD3+ T cells	33.14±11.97	29.26±13.08	29.85±11.30	24.19±9.95	
CD4+ T cells	36.64±16.36	30.96±14.71	31.55±11.97	23.94±11.28	<0.04 NP vs.3rd
CD8+ T cells	29.47±12.65	26.44±12.46	26.98±11.90	23.50±11.26	
NK cells	36.12±21.20	33.12±22.14	37.77±21.58	25.72±17.11	
NK^{dim} cells	35.74±20.58	32.59±21.98	36.72±20.73	24.65±16.45	
NK^{bright} cells	40.28±26.19	37.36±26.34	45.09±32.24	36.05±26.82	
NKT-like cells	60.45±18.60	67.22±12.49	62.78±15.24	52.53±17.58	<0.03 1st vs.3rd

Statistical comparisons were made in R using one-way ANOVA tests. The results were presented as the mean value ± SD. Differences were considered statistically significant for p-values ≤0.05. NP: Non-pregnant.

Supplementary Table S3. Surface expression of CD155 receptor (%) in peripheral blood mononuclear cell populations throughout pregnancy and in non-pregnant women.

	Non-pregnant	1st trimester	2nd trimester	3rd trimester	p-value
**Classical monocytes**	93.12±6.60	87.64±2.07	88.12±2.57	83.19±2.45	<0.03 NP vs.3rd
**Intermediater monocytes**	85.30±2.24	79.22±2.92	82.23±2.99	79.55±2.50	
**Non-classical monocytes**	74.04±2.61	69.19±2.47	71.27±3.22	71.91±2.79	
**CD3+ T cells**	0.45±0.08	0.49±0.10	0.59±0.12	0.30±0.05	
**CD4+ T cells**	0.22±0.04	0.26±0.06	0.29±0.08	0.14±0.03	
**CD8+ T cells**	0.55±0.13	0.64±0.13	0.81±0.20	0.46±0.10	
**NK cells**	1.03±0.14	1.52±0.20	1.29±0.13	1.44±0.19	
**NK^{dim} cells**	0.94±0.14	1.51±0.22	1.20±0.12	1.48±0.21	
**NK^{bright} cells**	2.34±0.28	1.74±0.23	1.73±0.27	1.20±0.16	
**NKT-like cells**	2.01±0.44	4.00±0.70	2.52±0.35	3.09±0.46	<0.02 NP vs. 1st

Statistical comparisons were made in R using one-way ANOVA tests. The results were presented as the mean value ± SD. Differences were considered statistically significant for p-values ≤0.05. NP: Non-pregnant.

Supplementary Table S4. Surface expression of CD112 receptor (%) in peripheral blood mononuclear cell populations throughout pregnancy and in non-pregnant women.

	Non-pregnant	1st trimester	2nd trimester	3rd trimester	p-value
**Classical monocytes**	45.99±4.65	35.41±2.63	39.57±4.15	37.78±2.79	
**Intermediate monocytes**	61.28±5.18	56.52±3.71	58.23±4.37	55.68±2.38	

**Non-classical monocytes**	48.55±4.46	46.82±3.10	47.82±3.39	48.78±2.66	
**CD3+ T cells**	3.88±0.51	4.68±0.74	5.44±0.62	5.36±0.68	
**CD4+ T cells**	3.22±0.48	3.96±0.62	4.73±0.56	4.86±0.69	
**CD8+ T cells**	4.25±0.56	5.05±0.81	5.60±0.75	5.72±0.69	
**NK cells**	4.76±0.62	5.95±0.75	6.77±0.74	7.12±0.74	
**NK^{dim} cells**	4.50±0.53	6.32±0.78	7.18±0.75	7.65±0.83	<0.03 NP vs.2nd<br><0.04 NP vs.3rd
**NK^{bright} cells**	3.25±0.60	3.15±0.48	3.97±0.60	3.63±0.52	
**NKT-like cells**	8.76±1.16	12.92±1.89	10.98±1.18	14.02±1.77	

Statistical comparisons were made in R using one-way ANOVA tests. The results were presented as the mean value ± SD. Differences were considered statistically significant for p-values ≤0.05. NP: Non-pregnant.
